# Evaluation of the Antimicrobial Activity of 20 Essential Oils and Their Combinations on Bacterial and Fungal Strains

**DOI:** 10.7759/cureus.79499

**Published:** 2025-02-23

**Authors:** Nihal Ezzariga, Abdellah Moukal, Ali Asdadi, Zohra Lemkhente, Fatima Moustaoui, Abderrazak Kaaya, Mohamed Aghrouch

**Affiliations:** 1 Microbiology, Faculty of Medicine and Pharmacy, Ibn Zohr University, Agadir, MAR; 2 Cellular Biology and Molecular Genetics, Faculty of Sciences, Ibn Zohr University, Agadir, MAR; 3 Pharmacology, Amadal Pharma Company, Agadir, MAR; 4 Infectious Disease, Faculty of Medicine and Pharmacy, Ibn Zohr University, Agadir, MAR; 5 Microbiology, Hassan II Regional Hospital, Agadir, MAR; 6 Medical Biology, Hassan II Regional Hospital, Agadir, MAR

**Keywords:** antimicrobial activity, bacterial strains, candida, escherichia coli, essential oils, fungal strains, pseudomonas aeruginosa, staphylococcus aureus

## Abstract

Introduction: Growing antibiotic resistance is driving the search for natural alternatives, such as essential oils (EOs), which are known for their antimicrobial properties. This study evaluated the antimicrobial efficacy of 20 EOs on bacterial and fungal strains and the impact of their combinations.

Materials and methods: Twenty EOs were selected to evaluate their antimicrobial potential in vitro. Stock solutions were prepared and tested on three bacterial strains (*Staphylococcus aureus, Escherichia coli*, and *Pseudomonas aeruginosa) *and three yeast strains of the genus *Candida*. The antimicrobial activity of EOs was initially evaluated by aromatogram, making it possible to classify oils according to their effectiveness. The sensitivity of microorganisms to EOs was then determined by measuring the minimum inhibitory concentration (MIC) and minimum bactericidal concentration (MBC). EO combination tests were also performed to detect synergistic or antagonistic effects.

Results: Tests revealed that *Origanum vulgare, Cymbopogon citratus,* and *Thymus leptobotrys* were particularly effective against Gram-positive bacteria and yeasts, while others, such as *Citrus limon*, showed negligible activity. The combinations of oils generally produced indifferent or antagonistic effects, especially against *P. aeruginosa.*

Conclusion: The results could open new perspectives for natural antimicrobial treatments, thus contributing to the fight against antibiotic resistance. In addition, the study will highlight the importance of judicious use of EO combinations to avoid interactions that could compromise their effectiveness.

## Introduction

The increasing resistance of bacteria to conventional antibiotics has become one of the most pressing threats to global public health today. According to the World Health Organization (WHO), the emergence and spread of resistant bacteria reduce the effectiveness of available treatments and lead to increased morbidity, mortality, and healthcare costs. Infections that were once easily treatable, such as urinary tract infections, pneumonia, and septicemia, are becoming increasingly difficult to control. This global health crisis is further exacerbated in the field of fungal infections due to the growing resistance of certain pathogenic fungi, such as *Candida auris*, to conventional antifungal agents [1].

In the face of this alarming situation, the search for innovative and effective solutions has become a priority for the scientific and medical community. Among the strategies being explored, the use of natural compounds derived from medicinal plants offers a promising perspective. In particular, essential oils (EOs), extracted from aromatic plants, have attracted attention due to their well-documented antimicrobial properties, recognized for centuries in various medicinal traditions [1]. These oils, primarily obtained through steam distillation or cold pressing, contain a multitude of bioactive compounds, including terpenes, phenols, and aldehydes, known for their ability to inhibit the growth of numerous pathogens [2].

In this context, the present study aims to evaluate the antibacterial and antifungal efficacy of 20 EOs extracted from medicinal plants. The objective is not only to identify the most promising oils but also to explore the effects of their combinations to maximize their antimicrobial activity. This research seeks to contribute to the development of new natural therapeutic approaches that can potentially address the antimicrobial resistance crisis while offering sustainable and environmentally friendly solutions.

## Materials and methods

The inclusion and exclusion criteria are as follows: Twenty species of aromatic and medicinal plants, originating from the Souss Massa region, were selected due to their traditional use in the treatment of certain infections. The EOs used in the study must be sourced from commercial suppliers, in accordance with the defined methodology, and come from the same supplier to ensure a consistent comparison of antimicrobial activities. The study is limited to the following reference bacterial strains: *Staphylococcus aureus* ATCC29213, *Escherichia coli *ATCC35218, and *Pseudomonas aeruginosa* ATCC27853, all standardized for microbiological testing, as well as *Candida* species from pathological samples processed in the medical mycology laboratory.

Twenty plant species from the aromatic and medicinal flora of the Souss Massa region (Table 1) were selected to assess their antimicrobial potential in vitro. The EOs used in the study were commercially sourced from a local herbalist (obtained by distillation) and stored at a temperature of +5°C (refrigerated). The solutions had a storage duration of three months, with a stability period of one year. They were protected from light and stored in tinted glass containers. For the preparation of EO stock solutions (50 mg/mL), each oil was dissolved in sterile Mueller-Hinton broth (MHB) for bacteria and Sabouraud dextrose broth (SDB) for yeasts, with 0.2% agar as an emulsifier. The solutions were then refrigerated at +5°C until use. The antimicrobial activity of the EOs was tested against three reference bacterial strains - *S. aureus *ATCC29213, *E. coli *ATCC35218, and *P. aeruginosa* ATCC27853 - and three fungal strains of the genus *Candida* (*C. albicans*, *C. glabrata*, and *C. tropicalis*), obtained from the Laboratory of Parasitology, Mycology, and Microbiology. The bacterial and fungal strains were revived and subcultured on nutrient agar (Mueller-Hinton agar (MHA) for bacteria and Sabouraud agar (SA) for yeasts), with microbial concentrations of the inoculum adjusted to 0.5 McFarland, corresponding to 10⁸ CFU/mL.

**Table 1:** Twenty plant species selected from the aromatic and medicinal flora of the Souss Massa region

Scientific name	Vernacular name	Local name	Family
Artemisia herba-alba	Artemisia	الشيح	Compositae
Citrus limon	Lemon	الحامض	Rutaceae
Citrus sinensis	Orange	الليمون	Rutaceae
Cupressus sempervirens	Sypresses	السرو	Cupressaceae
Cymbopogon citratus	Lemongrass	اللويزة الرومية	Poaceae
Laurus nobilis	Laurel leaf	عصى سيدنا موسى	Lauraceae
Lavandula angustifolia	Lavender	الخزامة	Lamiaceae
Lavandula dentata	Lavender	الخزامة	Lamiaceae
Melaleuca alternifolia	Tea tree	شجرة الشاي	Myrtaceae
Melaleuca quinquenervia	Niaouli	بلقاء خماسية العروق	Myrtaceae
Mentha piperita	Peppermint	النعناع العبدي	Lamiaceae
Ocimum basilicum	Basil	الحبق	Lamiaceae
Origanum vulgare	Wild marjoram	الزعتر الزعتر	Lamiaceae
Pistacia atlantica	Atlas pistachio	تتكت	Anacardiaceae
Rosmarinus officinalis	Rosemary	الأزير	Lamiaceae
Syzygium aromaticum	Clove	القرنفل	Myrtaceae
Tetraclinis articulata	White cedar	العرعار	Cupressaceae
Thymus leptobotrys	Thym L	الزعيترة	Lamiaceae
Thymus satureioides	Thym S	الزعيترة	Lamiaceae
Zingiber officinale	Ginger	الزنجبيل	Zingiberaceae

The antibacterial activity of EOs was assessed using the disk diffusion method, where Whatman Grade 1 filter paper discs (≈180 µm thickness, 6 mm diameter) impregnated with 10 µL of the EO stock solution were placed on agar (MHA for bacteria or SA for yeasts) previously inoculated with a microorganism suspension. After incubation for 24 hours at 37°C, the inhibition zones formed were measured in millimeters, with a positive test indicated by the use of active antibiotics for each germ. Based on the results, the study was expanded to investigate the synergistic effect of five EOs with significant antibacterial activity.

The minimum inhibitory concentration (MIC) was determined using the liquid microdilution method. In the first well of each microplate, 100 µL of the EO stock solution was added, and the remaining wells were filled with 50 µL of sterile MHB. A semi-logarithmic dilution was performed by transferring 50 µL from the first well to the next, creating a series of decreasing concentrations. To each well (1 to 11), 50 µL of a bacterial or yeast suspension was added, with the last well containing 100 µL of BMH as a control. The microdilution method was performed on microplates in liquid Mueller-Hinton medium (MHM) for bacteria and in liquid SDM for yeasts. For each strain, an inoculum of 5 x 10^6^ CFU/mL for bacteria and 5 x 10^4^ CFU/mL for yeasts was used. The plates were incubated for 24 hours at 37°C with constant stirring, and the MIC was defined as the lowest concentration preventing any visible bacterial growth. To determine the minimum bactericidal or fungicidal concentration (MBC or MFC), 10 µL of the bacterial suspensions from wells with no visible growth were subcultured onto MHA for bacteria and SA for yeasts. The plates were incubated for 24 hours for bacteria and 48 hours for yeasts at 37°C, and the MBC or MFC was determined from the first well showing no bacterial growth. All the tests (solid medium diffusion, MIC, MBC, and MFC) were carried out in triplicate.

The data were entered and organized in an Excel spreadsheet (Microsoft® Corp., Redmond, WA, USA) to ensure a structured and coherent analysis. The mean and standard deviation were calculated based on three repeated trials for each test.

## Results

Antibacterial screening of EOs

The aromatogram made it possible to classify EOs according to their antimicrobial activity, evaluated by the diameters of the inhibition zones. The categories identified were high activity (20 mm diameter), moderate activity (14 mm diameter ≤ 20 mm), low activity (8 mm diameter ≤ 14 mm), and no activity (diameter < 8 mm) [3-5].

**Figure 1:**
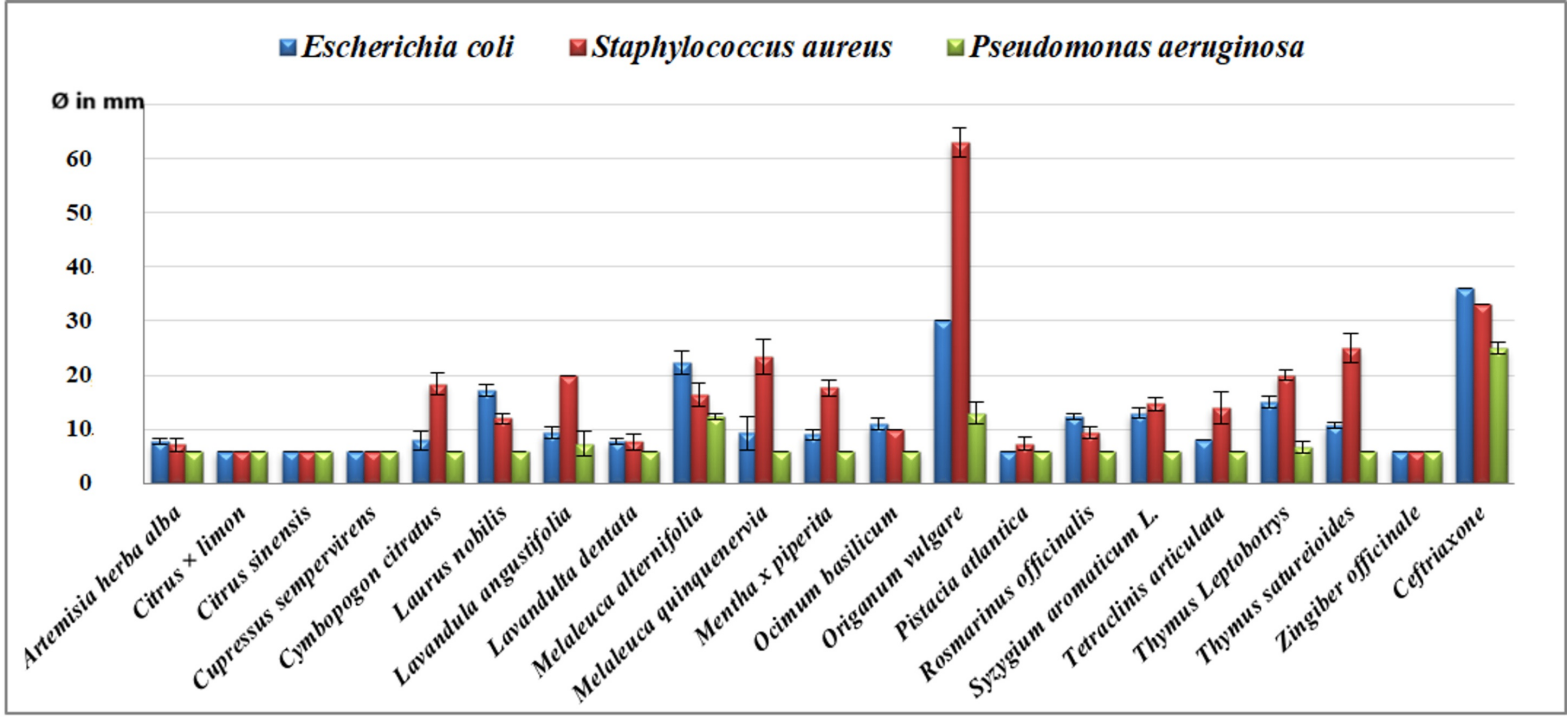
Essential oil inhibition diameter measured against the bacterial strains Escherichia coli, Pseudomonas aeruginosa, and Staphylococcus aureus (n = 3, Ø in mm)

**Figure 2:**
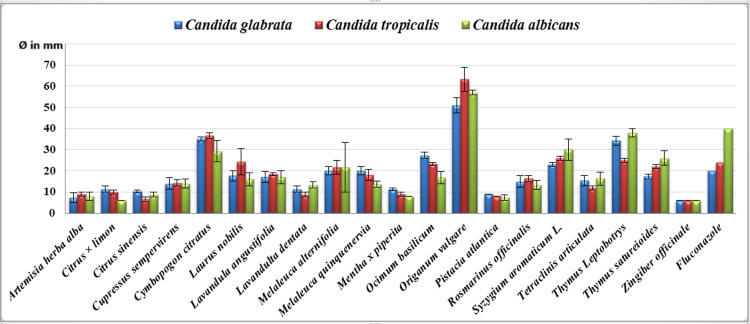
Essential oil inhibition diameter measured against Candida tropicalis, Candida glabrata, and Candida albicans species (n = 3, Ø in mm)

The EOs tested showed varying levels of antimicrobial activity. *Origanum vulgare* was distinguished by its high efficacy against all strains, especially yeast and Gram-positive bacteria. It was followed by *Cymbopogon citratus* and *Thymus leptobotrys*, which also showed notable efficacy against yeast. *Melaleuca alternifolia* showed moderate to intense activity against *S. aureus* and yeasts, although its efficacy was limited against Gram-negative bacteria. Other EOs, such as *Lavandula angustifolia*, *Cupressus sempervirens*, and *Pistacia atlantica*, exhibited more limited antibacterial activity. In contrast, oils such as *Citrus limon*, *Citrus sinensis,* and *Zingiber officinale* have not demonstrated a significant antibacterial effect, with inhibition diameters of less than 8 mm. The full results have been shown in Figures 1-2, as well as in Table 2.

**Table 2:** Inhibition diameter (in mm) indicating the antibacterial activity of essential oils by the disc diffusion method (n = 3, values expressed as mean ± SD) In a column, values followed by the same letter are not significantly different at p < 0.05 according to the Newman-Keuls test.

Plants	Gram-negative bacteria		Gram-positive bacteria	Yeasts		
	Escherichia coli	Pseudomonas aeruginosa	Staphylococcus aureus	Candida albicans	Candida glabrata	Candida tropicalis
Artemisia herba-alba	7.67±0.58ᵃᵇ	6ᵃ	7.17±1.26ᵃ	8±2ᵃᵇ	7.33±2ᵃᵇ	9±1ᵃᵇ
Cymbopogon citratus	8±1.73ᵃ	6ᵃ	18.33±2.08ᵃ	29.33±5ᵃᵇ	35±1ᶜᵈᵉ	37±1ᶜᵈᵉ
Citrus limon	6ᵃᵇ	6ᵃ	6ᶠ	6ᵉ	11.33±2ⁱ	10±1ʰ
Citrus sinensis	6ᵃ	6ᵃ	6ᵃ	8.67±1ᵃ	10.33±1ᵇᶜᵈ	7±1ᵃᵇᶜ
Cupressus sempervirens	6ᵃ	6ᵃ	6ᵃ	14±2ᵃᵇᶜ	14±3ᵇᶜ	14±2ᵃᵇ
Lavandula angustifolia	9.33±0.58ᵃᵇ	7.33±2.31ᵃ	20ᵇᶜ	17±3ᵃᵇᶜ	17±3ᵇᶜᵈ	18±1ᵃᵇ
Lavandula dentata	7.67±0.58ᶠ	6ᵃ	7.67±1.44ᵇᶜ	13.33±2ᵃᵇᶜ	11.33±2ᵉᶠ	9±1ᵍ
Laurus nobilis	17.17±1.26ᵇᶜ	6ᵃ	12±1ᶠ	16±3ᵇᶜ	17.67±3ᵉᶠ	24±6ᵉᶠ
Melaleuca alternifolia	22.33±2.08ᵍ	12.33±0.58ᵇ	16±2.08ᵈᵉᶠ	21.67±12ᶜᵈ	20±2ᶠᵍ	22±3ᶠᵍ
Mentha piperita	9±1ᵇᶜ	6ᵃ	17.67±1.53ᵍ	8ᵃᵇᶜ	11.33±1ᶠᵍ	9±1ᵉᶠ
Melaleuca quinquenervia	9.33±3.06ᵇᶜ	6ᵃ	23±3.21ᵉᶠ	13.67±2ᵃᵇ	20±2ᵇᶜᵈ	18±3ᵃᵇ
Ocimum basilicum	11±1ᶜᵈ	6ᵃ	10±1ᵃᵇ	16.67±3ᵇᶜ	27.33±2ʰ	23±1ᵍ
Origanum vulgare	30ʰ	13 ±2ᵇ	63±2.65ʰ	57±1ᵍ	51±4ʲ	63±6ⁱ
Pistacia atlantica	6ᵃ	6ᵃ	7.33±1.15ᵃ	7.33±1ᵃᵇ	9ᵃᵇ	8ᵃᵇ
Rosmarinus officinalis	12.33±0.58ᵈ	6ᵃ	9.33±1.15ᵃᵇ	13.33±2ᵃᵇᶜ	15±3ᶜᵈᵉ	16±2ᵈᵉ
Syzygium aromaticum	13±1ᵈ	6ᵃ	14.67±1.15ᶜᵈᵉ	30±5ᵉ	23±1ᵍ	26±1ᵍ
Tetraclinis articulata	7.67±0.29ᵃᵇ	6ᵃ	14±3ᶜᵈ	16.33±3ᵇᶜ	15.33±3ᵈᵉᶠ	12±1ᵇᶜᵈ
Thymus leptobotrys	15±1ᵉ	6.67±1.15ᵃ	20±1ᶠ	38±2ᶠ	34.33±2ⁱ	25±1ᵍ
Thymus satureioides	10.67±0.58ᶜᵈ	6ᵃ	25±2.65ᵍ	26±3ᵈᵉ	17.33±1ᵉᶠ	22±1ᶠᵍ
Zingiber officinale	6ᵃ	6ᵃ	6ᵃ	6ᵃ	6ᵃ	6ᵃ
Ceftriaxone (CRO)	36	25	33	-	-	-
Fluconazole	-	-	-	40	20	24

The results show that the EO of *O. vulgare* (30 mm, a) exhibits the highest antimicrobial activity against *E. coli*, significantly surpassing other oils such as *C. limon* (6 mm, d) and *Z. officinale* (6 mm, d), which showed no inhibition. Other oils, such as *Laurus nobilis* (17.17 mm, b) and *M. alternifolia* (22.33 mm, c), displayed significant activity but were statistically distinct from *Z. officinale* and *C. sinensis* (6 mm, d). The Newman-Keuls test confirmed that the differences between *O. vulgare* and the other oils are significant.

Regarding *P. aeruginosa*, *O. vulgare* (13 mm, a) and *M. alternifolia* (12.33 mm, a) exhibited moderate inhibition, while *C. limon* (6 mm, d), *Z. officinale *(6 mm, d), and *C. citratus* (6 mm, d) showed no significant activity. The test confirmed that no significant differences exist between these oils.

For *S. aureus*, *O. vulgare* (63 mm, a) demonstrated the highest inhibition, followed by *M. alternifolia* (16 mm, b) and *L. angustifolia* (20 mm, b). In contrast, *C. limon* (6 mm, d) and *C. citratus* (18.33 mm, c) showed lower inhibition levels. The test revealed significant differences between *O. vulgare* and the other oils.

For *C. albicans*, *O. vulgare* (57 mm, a) was the most effective, followed by *T. leptobotrys* (38 mm, b) and *C. citratus* (29.33 mm, c). *C. limon* (6 mm, d) and *Z. officinale* (6 mm, d) showed weak inhibition. The test confirmed that these differences are significant.

Finally,* O. vulgare *achieved the best results against *C. glabrata* (51 mm, a) and *C. tropicalis* (63 mm, a), while *T. leptobotrys* (25 mm, b) and *Syzygium aromaticum* (26 mm, b) exhibited lower inhibition. *Z. officinale* and *C. limon* showed no activity against these yeasts, as confirmed by the Newman-Keuls test.

EO combinations

The study of EO combinations against *P. aeruginosa* (Table 3) found that the effects were mainly antagonistic and indifferent.

**Table 3:** Effect of combinations of selected essential oils on Pseudomonas aeruginosa (n = 3, values expressed as mean ± SD)

Combination			Diameter (mm)	Effect of combinations
Melaleuca alternifolia	+	Lavandula angustifolia	7.66 ± 1.53	Antagonistic effect
Melaleuca alternifolia	+	Melaleuca quinquenervia	6	Antagonistic effect
Melaleuca alternifolia	+	Origanum vulgare	15.67 ± 2.08	Antagonistic effect
Melaleuca alternifolia	+	Thymus leptobotrys	8 ± 3.46	Antagonistic effect
Melaleuca quinquenervia	+	Lavandula angustifolia	8	Indifferent effect
Melaleuca quinquenervia	+	Origanum vulgare	14.67 ± 1.15	Antagonistic effect
Melaleuca quinquenervia	+	Thymus leptobotrys	6.67 ±1.15	Indifferent effect
Lavandula angustifolia	+	Origanum vulgare	13.33 ± 2.51	Indifferent effect
Lavandula angustifolia	+	Thymus leptobotrys	7.33 ± 1.528	Indifferent effect
Origanum vulgare	+	Thymus leptobotrys	14.67 ± 0.56	Antagonistic effect
Origanum vulgare	+	Zingiber officinale	6	Antagonistic effect
Origanum vulgare	+	Zingiber officinale	22	Antagonistic effect
Origanum vulgare	+	Ceftriaxone	25	Indifferent effect
Zingiber officinale	+	Ceftriaxone	24	Antagonistic effect

The combinations of *M. alternifolia *with *L. angustifolia*, *M. quinquenervia*, *O. vulgare*, and *T. leptobotrys* exhibit an antagonistic effect, with inhibition diameters ranging from 6 to 15.67 mm. This indicates that these combined EOs show reduced effectiveness compared to the individual use of each oil. The combinations of *M. quinquenervia* with *L. angustifolia* and *T. leptobotrys*, as well as *L. angustifolia* with *O. vulgare *and *T. leptobotrys*, exhibit indifferent effects, with moderate inhibition zones ranging from 6 to 14.67 mm, suggesting that no significant interaction occurs between these oils. A particular observation concerns the combination of *O. vulgare* with *Z. officinale*, which generates an inhibition diameter of 22 mm, suggesting enhanced effectiveness compared to other combinations. In summary, the majority of combinations show an antagonistic effect, while some show no notable effect, and one combination demonstrates particularly high efficacy.

Sensitivity of EOs: MIC and MBC

The MIC was determined only for EOs from plant species to which the tested strains showed high sensitivity, with an inhibition diameter equal to or greater than 15 mm (D ≥ 15 mm). The results are detailed in Tables 4-5.

**Table 4:** Minimum inhibitory concentrations (MIC) and minimum bactericidal concentrations (MBC) of essential oils from studied plant species (in mg/mL) In a column, values followed by the same letter are not significantly different at p < 0.05 according to the Newman-Keuls test.

Essential oils	Escherichia coli			Staphylococcus aureus		
	MIC	MBC	MBC/MIC	MIC	MBC	MBC/MIC
Cymbopogon citratus	Not tested	Not tested	Not tested	1.148ᵃᵇ	2.755ᵃ	2.4
Lavandula angustifolia	Not tested	Not tested	Not tested	3.223ᵇᶜ	6.447ᵇ	2
Laurus nobilis	5.588ᶜ	5.588ᶜ	1	Not tested	Not tested	Not tested
Melaleuca alternifolia	1.830ᵇ	1.830ᵇ	1	5.491ᶜᵈ	5.491ᵇ	1
Mentha piperita	Not tested	Not tested	Not tested	5.575ᶜᵈ	11.150ᶜ	2
Melaleuca quinquenervia	Not tested	Not tested	Not tested	7.365ᵈ	7.365ᵇ	1
Origanum vulgare	0.352ᵃ	0.352ᵃ	1	0.176ᵃ	0.352ᵃ	2
Thymus leptobotrys	0.662ᵃ	0.662ᵃ	1	0.662ᵃᵇ	1.324ᵃ	2
Thymus satureioides	Not tested	Not tested	Not tested	1.045ᵃᵇ	2.091ᵃ	2

**Table 5:** Minimum inhibitory concentrations (MIC) and minimum fungicidal concentrations (MFC) of essential oils from studied plant species (in mg/mL) In a column, values followed by the same letter are not significantly different at p < 0.05 according to the Newman-Keuls test.

Essential oils	Candida glabrata			Candida tropicalis			Candida albicans		
	MIC	MFC	MFC/MIC	MIC	MFC	MFC/MIC	MIC	MFC	MFC/MIC
Cymbopogon citratus	0.172ᵃ	0.344ᵃ	2	0.172ᵃ	0.172ᵃ	1	0.172ᵃ	0.172ᵃ	1
Lavandula angustifolia	3.223ᵇ	6.446ᶜ	2	3.223ᶜ	3.223ᵇ	1	3.223ᵃ	3.223ᵃ	1
Laurus nobilis	2.794ᵇ	2.794ᵃᵇᶜ	1	2.794ᶜ	2.794ᵇ	1	1.397ᵃ	2.794ᵃ	2
Mentha piperita	1.373ᵃᵇ	1.373ᵃᵇ	1	1.373ᵇ	2.745ᵇ	2	1.373ᵃ	2.745ᵃ	2
Melaleuca quinquenervia	0.172ᵃ	0.172ᵃ	1	0.172ᵃ	0.172ᵃ	1	0.69ᵃ	0.69ᵃ	1
Ocimum basilicum	5.756ᶜ	5.756ᵇᶜ	1	5.756ᵈ	5.756ᶜ	1	5.756ᵇ	5.756ᵇ	1
Origanum vulgare	0.088ᵃ	0.088ᵃ	1	0.088ᵃ	0.088ᵃ	1	0.088ᵃ	0.176ᵃ	2
Rosmarinus officinalis	5.675ᶜ	5.675ᵇᶜ	2	5.675ᵈ	5.675ᶜ	1	Not tested	Not tested	Not tested
Syzygium aromaticum	0.406ᵃ	0.406ᵃ	1	0.406ᵃ	0.406ᵃ	1	0.813ᵃ	0.813ᵃ	1
Tetraclinis articulata	2.905ᵇ	5.809ᵇᶜ	2	Not tested	Not tested	Not tested	2.905ᵃ	2.905ᵃ	2.905
Thymus leptobotrys	0.331ᵃ	0.331ᵃ	1	0.662ᵃᵇ	0.662ᵃ	1	0.331ᵃ	0.331ᵃ	1
Thymus satureioides	1.568ᵃᵇ	1.568ᵃᵇ	1	0.784ᵃᵇ	0.784ᵃ	1	0.784ᵃ	1.568ᵃ	2

The MIC was determined only for EOs that exhibited a strong inhibitory effect (D ≥ 15 mm). The results indicate that *O. vulgare* and *T. leptobotrys* have the highest antibacterial activity against both bacterial strains, with the lowest MIC and MBC values. Conversely, *L. nobilis* showed the weakest effect against *E. coli*, while *M. quinquenervia* and *Mentha piperita* were the least effective against *S. aureus.*

The MBC/MIC ratios suggest that most of the tested EOs have a bactericidal effect (≤4), with *O. vulgare* displaying the strongest activity. Statistical analysis (Newman-Keuls test, p < 0.05) highlights significant differences between the tested oils, further reinforcing the potential of *O. vulgare* and *T. leptobotrys* as promising natural antibacterial agents.

*C. citratus* exhibits strong antifungal activity, with low MICs of 0.172 mg/mL for all three *Candida* species. The MFCs range from 0.172 mg/mL for *C. albicans* to 0.344 mg/mL for *C. glabrata* and *C. tropicalis*, showing consistent inhibitory and fungicidal effects across species without significant statistical differences. *L. angustifolia* is less effective, requiring MICs of 3.223 mg/mL and MFCs from 3.223 mg/mL for *C. albicans* to 6.446 mg/mL for *C. glabrata* and *C. tropicalis*, indicating the need for higher concentrations for efficacy. *L. nobilis* shows moderate activity, with MICs of 1.397 mg/mL for *C. albicans* and 2.794 mg/mL for *C. glabrata* and *C. tropicalis*. *M. piperita* is moderately effective, with MICs of 1.373 mg/mL for *C. glabrata* and *C. albicans*, though higher concentrations are needed for *C. tropicalis*. *M. quinquenervia* demonstrates strong antifungal effects, with MICs of 0.172 mg/mL for all species, though the MFC for *C. albicans* is higher (0.69 mg/mL). *O. vulgare* stands out with low MICs (0.088 mg/mL for all species), but a significantly higher MFC for *C. albicans* (0.176 mg/mL). Other EOs, including *Ocimum basilicum*, *Rosmarinus officinalis*, *S. aromaticum*, *Tetraclinis articulata*, *T. leptobotrys*, and *T. satureioides*, showed varying MIC and MFC values with significant statistical differences.

## Discussion

The results revealed the potential of EOs to overcome specific microbial resistances. Oils such as *O. vulgare, M. alternifolia, *and *C. citratus *demonstrated promising antimicrobial activity, particularly against *S. aureus* and yeasts of the genus *Candida*. However, Gram-negative bacteria exhibited greater resistance.

The results obtained are consistent with those of previous research. Regarding Gram-negative bacteria, *O. vulgare* demonstrated potent activity against *E. coli*, confirming the observations of Derradji et al. [3]. *M. alternifolia, L. nobilis*, and *T. leptobotrys* showed moderate activity, aligning with Thomsen et al. studies, respectively [6-8]. Conversely, *R. officinalis*, *M. quinquenervia*, *M. piperita*, and *O. basilicum* exhibited low activity, corroborated by other research [4,9].

Regarding *P. aeruginosa*, *O. vulgare* and *M. alternifolia *showed moderate activity, corresponding to previously observed results [3,6]. *L. angustifolia* revealed low activity, in line with earlier observations [10]. The other tested EOs did not demonstrate significant activity against this bacterium, likely due to its outer membrane and efflux systems, which reduce the efficacy of EOs.

In contrast, Gram-positive bacteria are more sensitive. *O. vulgare *and *T. satureioides* demonstrated potent activity against *S. aureus*, confirmed by previous studies [3]. The moderate values observed for *M. alternifolia*, *T. leptobotrys*, *C. citratus*, and *L. angustifolia* are also supported by other studies [9,11,12]. As reported in prior studies, *R. officinalis *and *O. basilicum* exhibited low activity [9,13].

Due to their hydrophilic outer membrane, Gram-negative bacteria are less sensitive to EOs. This barrier limits the diffusion of active compounds, making these bacteria more resistant. Additionally, *P. aeruginosa* possesses intrinsic resistance mechanisms, including efflux pumps and the ability to form biofilms, further reducing the efficacy of EOs [14,15].

Regarding yeasts, *O. vulgare, C. citratus,* and *T. leptobotrys* showed potent activity against *C. albicans, C. glabrata*, and *C. tropicalis*, as demonstrated by studies by Giordani et al. and Manohar et al. [16,17]. *M. alternifolia, M. quinquenervia,* and *O. basilicum* exhibited moderate activity against *C. albicans*, consistent with findings from the studies by Fedoul et al., Noumi et al., and Silva et al. [18-20].

Some EOs, such as *Artemisia herba-alba, C. limon, C. sinensis, P. atlantica, *and *Z. officinale*, showed no significant antimicrobial activity, as confirmed by other studies [21,22]. Similarly, *C. sempervirens* exhibited no antibacterial activity and limited antifungal activity [9].

EO combinations

The combinations of EOs tested against *P. aeruginosa *primarily demonstrated antagonistic or indifferent effects, with no notable synergy. This could be due to the richness of these oils in compounds such as thymol, carvacrol, linalool, terpenes, and cineole, which individually possess potent antimicrobial properties. However, their interactions may sometimes impair their effectiveness. For instance, combinations of thymol with carvacrol or carvacrol with myrcene and p-cymene showed diminished antimicrobial effects compared to their individual use [23,24]. Additionally, linalool may reduce the efficacy of mixtures by decreasing the bioavailability of active compounds [1]. Terpenes and cineole may also affect effectiveness by altering the solubility of compounds or interfering with bacterial defense mechanisms [25]. It is also possible that the intrinsic resistance of *P. aeruginosa* masks the synergistic effects these combinations might exert on other, more sensitive microorganisms.

Sensitivity of EOs

This study evaluated the effectiveness of EOs against *E. coli, S. aureus*, and three species of *Candida* by measuring their inhibitory, bactericidal, and fungicidal capacities. Regarding *E. coli*, the EOs of *O. vulgare* and *T. leptobotrys* showed high inhibitory activities (low MIC values), confirming previous results [26,8]. *M. alternifolia* also demonstrated good antibacterial activity, corroborated by other studies [6]. Against *S. aureus*, the EOs of *O. vulgare* and *T. leptobotrys* also exhibited moderate activity, as reported in earlier studies [12,27]. Conversely, *M. piperita* and *M. quinquenervia* required higher concentrations for bactericidal action, suggesting lower efficacy [28].

The antibacterial properties of these EOs are primarily attributed to their content of bioactive compounds, such as carvacrol and thymol, which disrupt the bacterial cell membranes, leading to leakage of fundamental constituents and cell death [1,14,25]. *O. vulgare *and *T. leptobotrys *have lower MIC values and an MBC/MIC ratio of 1, indicating effective bactericidal action.

Regarding the antifungal effect, *O. vulgare* showed potent activity against yeasts of the genus *Candida*, attributed to its richness in carvacrol and thymol [29]. *C. citratus *and *M. quinquenervia* also displayed good antifungal activity due to their content of citral and terpinen-4-ol [20,30]. EOs such as *S. aromaticum* and *T. leptobotrys* showed moderate efficacy [31,32], while *L. nobilis, M. piperita*, and *T. articulata *demonstrated limited effectiveness.

The antifungal efficacy of these EOs largely depends on their chemical composition and the mechanisms of action of the active compounds. EOs rich in carvacrol, thymol, eugenol, and citral, such as *O. vulgare, S. aromaticum, *and *T. leptobotrys*, disrupt yeasts' cellular processes, including protein synthesis and membrane permeability [28,33]. In contrast, EOs like *O. basilicum* and *L. angustifolia* show lower effectiveness, likely due to their low concentration of active antifungal compounds [34].

The limitations of this study include the variability in the chemical composition of EOs, which depends on factors such as geographical origin, cultivation conditions, and extraction methods, thereby influencing their antimicrobial properties and the reproducibility of the results. Additionally, the lack of standardization in testing and the limited number of microbial strains studied reduce the scope of the conclusions, and an evaluation of a larger sample, including resistant strains, would be necessary to strengthen the validity of the results.

## Conclusions

Certain EOs show promising potential for the development of new antimicrobial agents. However, potential antagonistic effects must be considered when combining these oils to avoid interactions that could diminish their efficacy. Furthermore, additional research is needed to clarify their mechanisms of action and interactions. Finally, clinical studies are crucial to validate their effectiveness, safety, and tolerability in order to optimize their therapeutic use.

## References

[1] Bassolé IH, Juliani HR (2012). Essential oils in combination and their antimicrobial properties. Molecules.

[2] Chabenat H (2017). In vitro potentiality of 10 essential oils, alone or in combination, in the treatment of cutaneous bacterial infections [Thesis in French]. Limoges.

[3] Leila D, Ouided S, Youcef H (2020). Evaluation of the antibacterial activity of three essential oils extracted from plants used in traditional medicine in Algeria (Salvia officinalis L, Melissa officinalis L, and Origanum vulgare L). GSC Biol Pharm Sci.

[4] Diallo A, Tine Y, Diop A (2020). Chemical composition and antibacterial activity of essential oil of Melaleuca quinquenervia (Cav.) S.T. Blake (Myrtaceae). Asian J Appl Chem Res.

[5] Mutai C, Bii C, Vagias C, Abatis D, Roussis V (2009). Antimicrobial activity of Acacia mellifera extracts and lupane triterpenes. J Ethnopharmacol.

[6] Thomsen PS, Jensen TM, Hammer KA, Carson CF, Mølgaard P, Riley TV (2011). Survey of the antimicrobial activity of commercially available Australian tea tree (Melaleuca alternifolia) essential oil products in vitro. J Altern Complement Med.

[7] Caputo L, Nazzaro F, Souza LF (2017). Laurus nobilis: composition of essential oil and its biological activities. Molecules.

[8] Oubihi A, Ouryemchi I, Nounah I, Tarfaoui K, Harhar H, Ouhssine M, Guessous Z (2020). Chemical composition, antibacterial and antifungal activities of Thymus leptobotrys Murb essential oil. Adv Tradit Med.

[9] Bouftila B, Abdessadek M, Ben-Saghroune H, Sbihi K, Ouahmane L, Amri O, El Amraoui B (2024). Antimicrobial activities of essential oils of plants species from Morocco against some microbial strains. Rocz Panstw Zakl Hig.

[10] Hossain S, Heo H, De Silva BC, Wimalasena SH, Pathirana HN, Heo GJ (2017). Antibacterial activity of essential oil from lavender (Lavandula angustifolia) against pet turtle-borne pathogenic bacteria. Lab Anim Res.

[11] Zhang X, Guo Y, Guo L, Jiang H, Ji Q (2018). In vitro evaluation of antioxidant and antimicrobial activities of Melaleuca alternifolia essential oil. Biomed Res Int.

[12] Razzouk S, Mazri MA, Jeldi L, Mnasri B, Ouahmane L, Alfeddy MN (2022). Chemical composition and antimicrobial activity of essential oils from three Mediterranean plants against eighteen pathogenic bacteria and fungi. Pharmaceutics.

[13] Djelloul R, Mokrani K, Hacini N (2017). Study of the antibacterial activity of the extract from the essential oil of Eucalyptus globulus and Rosmarinus officinalis on three bacterial strains. Int J Appl Environ Sci.

[14] Poole K (2005). Efflux-mediated antimicrobial resistance. J Antimicrob Chemother.

[15] Hall CW, Mah TF (2017). Molecular mechanisms of biofilm-based antibiotic resistance and tolerance in pathogenic bacteria. FEMS Microbiol Rev.

[16] Giordani R, Regli P, Kaloustian J, Mikaïl C, Abou L, Portugal H (2004). Antifungal effect of various essential oils against Candida albicans. Potentiation of antifungal action of amphotericin B by essential oil from Thymus vulgaris. Phytother Res.

[17] Manohar V, Ingram C, Gray J, Talpur NA, Echard BW, Bagchi D, Preuss HG (2001). Antifungal activities of origanum oil against Candida albicans. Mol Cell Biochem.

[18] Fedoul FF, Meddah B, Larouci M (2022). Medicinal applications chemical compositions and biological effects of Algerian Ocimum basilicum L.var Genovese with the conversion of experimental doses to humans. J Appl Biotech Rep.

[19] Noumi E, Mejdi S, Hajlaoui H, Trabelsi N, Ksouri R, Valentin E, Bakhrouf A (2011). Chemical composition, antioxidant and antifungal potential of Melaleuca alternifolia (Tea Tree) and Eucalyptus globulus essential oils against oral Candida species. J Med Plant Res.

[20] Silva RAD, Antonieti FMPM, Röder DVDB, Pedroso RDS (2021). Essential oils of Melaleuca, citrus, Cupressus, and Litsea for the management of infections caused by Candida species: a systematic review. Pharmaceutics.

[21] Amor G, Caputo L, La Storia A, De Feo V, Mauriello G, Fechtali T (2019). Chemical composition and antimicrobial activity of Artemisia herba-alba and Origanum majorana essential oils from Morocco. Molecules.

[22] Chalal C, Salmi O (2018). Study of the antimicrobial activity of essential oils of Citrus sinensis and Mentha x piperita L. in combination with antibiotics [Thesis in French]. Université Mouloud Mammeri.

[23] Gallucci MN, Oliva M, Casero C, Dambolena J, Luna A, Zygadlo J, Demo M (2009). Antimicrobial combined action of terpenes against the food‐borne microorganisms Escherichia coli, Staphylococcus aureus and Bacillus cereus. Flavour Fragr J.

[24] Hammer KA, Carson CF, Riley TV (1999). Antimicrobial activity of essential oils and other plant extracts. J Appl Microbiol.

[25] Mulyaningsih S, Sporer F, Zimmermann S, Reichling J, Wink M (2010). Synergistic properties of the terpenoids aromadendrene and 1,8-cineole from the essential oil of Eucalyptus globulus against antibiotic-susceptible and antibiotic-resistant pathogens. Phytomedicine.

[26] Kaloustian J, Chevalier J, Mikail C, Martin M, Abou L, Vergnes MF (2008). Study of six essential oils: chemical composition and antibacterial activity [Article in French]. Phytotherapie.

[27] Djouhaina MH, Hanane MB (2021). Evaluation of the antibacterial potential of bioactive plant extracts [Dissertation in French]. Evaluation du potentiel antibactérien des extraits bioactifs des plantes.

[28] Singh R, Shushni MAM, Belkheir A (2015). Antibacterial and antioxidant activities of Mentha piperita L. Arab J Chem.

[29] Pedroso RDS, Balbino BL, Andrade G (2019). In vitro and in vivo anti-Candida spp. activity of plant-derived products. Plants (Basel).

[30] Tyagi AK, Malik A (2010). Liquid and vapour-phase antifungal activities of selected essential oils against Candida albicans: microscopic observations and chemical characterization of Cymbopogon citratus. BMC Complement Altern Med.

[31] Asdadi A, Alilou H, Akssira M (2014). Chemical composition and anticandidal effect of three Thymus species essential oils from southwest of Morocco against the emerging nosocomial fluconazole-resistant strains. J Biol Agric Healthc.

[32] Loukili EH, Ouahabi S, Elbouzidi A (2023). Phytochemical composition and pharmacological activities of three essential oils collected from eastern Morocco (Origanum compactum, Salvia officinalis, and Syzygium aromaticum): a comparative study. Plants (Basel).

[33] Nazzaro F, De Martino L, Fratianni F, De Feo V (2020). Chapter 49 - essential oils from Mediterranean aromatic plants. The Mediterranean Diet (Second Edition).

[34] Burt S (2004). Essential oils: their antibacterial properties and potential applications in foods - a review. Int J Food Microbiol.

